# Postpunktioneller Kopfschmerz nach rückenmarknahen Anästhesieverfahren: Inzidenz und Risikofaktoren

**DOI:** 10.1007/s00101-020-00846-y

**Published:** 2020-09-16

**Authors:** J. Weinrich, C. von Heymann, A. Henkelmann, F. Balzer, A. Obbarius, P. V. Ritschl, C. Spies, P. Niggemann, L. Kaufner

**Affiliations:** 1 Klinik für Anästhesiologie mit Schwerpunkt operative Intensivmedizin (CCM, CVK), Charité – Universitätsmedizin Berlin, corporate member of Freie Universität Berlin, Humboldt-Universität zu Berlin, and Berlin Institute of Health, Augustenburger Platz 1, 13353 Berlin, Deutschland; 2 Klinik für Anästhesie, Intensivmedizin, Notfallmedizin und Schmerztherapie, Vivantes Klinikum im Friedrichshain, Landsberger Allee 49, Berlin, 10249 Deutschland; 3 Medizinische Klinik mit Schwerpunkt Psychosomatik, Zentrum für Innere Medizin und Dermatologie, Charité – Universitätsmedizin Berlin, corporate member of Freie Universität Berlin, Humboldt-Universität zu Berlin, and Berlin Institute of Health, Augustenburger Platz 1, Berlin, 13353 Deutschland; 4 Chirurgische Klinik, Campus Charité Mitte/Campus Virchow-Klinikum, Charité – Universitätsmedizin Berlin, corporate member of Freie Universität Berlin, Humboldt-Universität zu Berlin, and Berlin Institute of Health, Augustenburger Platz 1, Berlin, 13353 Deutschland

**Keywords:** Kombinierte Spinal-Epiduralanästhesie, Blutpatch, Regionalanästhesie, Spinalanästhesie, Risikofaktoren, Combined Spinal-Epidural-Anesthesia, Length of Hospital Stay, Bloodpatch, Spinal anesthesia, Risk factors

## Abstract

**Hintergrund/Ziel der Arbeit:**

Der postpunktionelle Kopfschmerz (PKS) ist eine Komplikation nach rückenmarknahen Verfahren (RA) mit erheblichem Krankheitswert. Ziel der Untersuchung war es, die Inzidenz des PKS in 2 großen operativen Kollektiven zu untersuchen, mögliche Risikofaktoren zu identifizieren und den Einfluss auf die Krankenhausverweildauer zu untersuchen.

**Material und Methoden:**

In einer retrospektiven Analyse des Zeitraums 2010–2012 wurden 341 unfallchirurgische (UCH) und 2113 geburtsmedizinische (GEB) Patient*innen nach Spinalanästhesie (SPA) analysiert. In der statistischen Auswertung (SPSS-23) kamen univariate Analysen mittels Mann-Whitney-U-, Chi^2^- und Student’s t‑Test sowie logistische Regressionsanalysen zur Anwendung.

**Ergebnisse:**

Die Inzidenz des PKS betrug in der UCH-Gruppe 5,9 % und in der GEB-Gruppe 1,8 %. Patient*innen mit PKS in der UCH wiesen ein jüngeres Patientenalter (38 vs. 47 Jahre, *p* = 0,011), einen geringeren BMI (23,5 vs. 25,2, *p* = 0,037) sowie ein niedrigeres Köpergewicht (70,5 kg vs. 77 kg, *p* = 0,006) als Patient*innen ohne PKS auf. Dabei konnten das Alter mit einer „odds ratio“ (OR 97,5 % Konfidenzintervall [KI]) von 0,963 (97,5% KI 0,932–0,991, *p* = 0,015) und das Köpergewicht mit einer OR von 0,956 (97,5 % KI 0,920–0,989, *p* = 0,014) als unabhängige Risikofaktoren für die Entstehung eines PKS identifiziert werden. In der GEB wies die SPA eine höhere Inzidenz des PKS auf als die kombinierte Spinalepiduralanästhesie (CSE) (8,6 % vs. 1,2 %, *p* < 0,001). Dabei erwies sich das Verfahren mit einer OR von 0,049 (97,5 % KI 0,023–0,106, *p* < 0,001) als unabhängiger Risikofaktor für die Entstehung eines PKS. In beiden Gruppen war der PKS mit einem verlängerten Krankenhausaufenthalt assoziiert (UCH-Gruppe 4 vs. 2 Tage, *p* = 0,001; GEB-Gruppe 6 vs. 4 Tage, *p* < 0.0005).

**Diskussion:**

Die Inzidenz des PKS nach SPA/CSE war in unserer Untersuchung in den beschriebenen Patientengruppen unterschiedlich, mit einem deutlich höheren Anteil in der UCH-Gruppe. Alter, Konstitution und Verfahren waren hinweisgebende Risikofaktoren eines PKS. In Anbetracht der funktionellen Einschränkungen (Mobilisation, Versorgung des Neugeborenen) und des verlängerten Krankenhausaufenthalts, sollten zukünftige Studien eine frühe Behandlung des PKS untersuchen.

## Abstract (en)

**Background/objective:**

Postdural puncture headache (PDPH) is a severe complication after spinal anesthesia. The aim of this study was to investigate the incidence of PDPH in two different operative cohorts and to identify risk factors for its occurrence as well as to analyze its influence on the duration of hospital stay.

**Material and methods:**

In a retrospective study over a period of 3 years (2010–2012), 341 orthopedic surgery (ORT) and 2113 obstetric (OBS) patients were evaluated after spinal anesthesia (SPA). Data were statistically analyzed using (SPSS-23) univariate analyses with the Mann-Whitney U‑test, χ^2^-test and Student’s *t-*test as well as logistic regression analysis.

**Results:**

The incidence of PDPH was 5.9% in the ORT cohort and 1.8% in the OBS cohort. Patients with PDPH in the ORT cohort were significantly younger (median 38 years vs. 47 years, *p* = 0.011), had a lower body weight (median 70.5 kg vs. 77 kg, *p* = 0.006) and a lower body mass index (median 23.5 vs. 25.2, *p* = 0.037). Body weight (odds ratio (97.5 % Confidence Intervall [CI]), OR 0.956: 97.5% CI 0.920–0.989, *p* = 0.014) as well as age (OR 0.963: 97.5% CI 0.932–0.991, *p* = 0.015) were identified as independent risk factors for PDPH. In OBS patients, PDPH occurred more frequently after spinal epidural anesthesia than after combined spinal epidural anesthesia (8.6% vs. 1.2%, *p* < 0.001) and the type of neuraxial anesthesia was identified as an independent risk factor for PDPH (OR 0.049; 97.5% CI 0.023–0.106, *p* < 0.001). In both groups the incidence of PDPH was associated with a longer hospital stay (ORT patients 4 days vs. 2 days, *p* = 0.001; OBS patients 6 days vs. 4 days, *p* < 0.0005).

**Conclusion:**

The incidence of PDPH was different in the two groups with a higher incidence in the ORT but considerably lower than in the literature. Age, constitution and type of neuraxial anesthesia were identified as risk factors of PDPH. Considering the functional imitations (mobilization, neonatal care) and a longer hospital stay, future studies should investigate the impact of an early treatment of PDPH.

Der postpunktionelle Kopfschmerz (PKS) ist eine häufige und leidvolle Komplikation rückenmarknaher Anästhesieverfahren (RA). Dabei schwankt seine Inzidenz in Abhängigkeit von der Patientengruppen bzw. der Art des operativen Eingriffs und verschiedenen verfahrens-, struktur- und patientenbedingten Risikofaktoren. Ziel dieser Untersuchung war es, die Inzidenz des PKS in 2 Patientengruppen zu ermitteln und zu vergleichen und somit spezifische Risikofaktoren für die Entstehung eines PKS sowie dessen Auswirkungen auf die Krankenhausverweildauer zu untersuchen.

## Hintergrund und Fragestellung

Der PKS nach RA ist eine häufige und für die Betroffenen oftmals leidvolle Komplikation. Pathophysiologisch wird einerseits eine Leckage im Liquorsystem angenommen, die nach einer verfahrensbedingten (Spinalanästhesie, SPA; kombinierte Spinalepiduralanästhesie, CSE) oder akzidentellen Duraperforation (Periduralanästhesie, PDA) eintritt [13] und zu einer als ursächlich angenommenen, unterdruckvermittelten, meningealen Reizung und Dilatation intrakranieller Gefäße (Monro-Kellie-Theorie) führt [20]. Andererseits können punktionstraumabedingte Gewebeanteile im Liquor zu einer meningealen Reizung führen, ohne dass es hierfür einer Druckverschiebung im Liquorsystem bedarf [26]. Maßgeblich für die Definition des PKS sind die Kriterien der International Headache Society, die v. a. eine Lageabhängigkeit des PKS im Sinne einer Zunahme der Symptomatik innerhalb von 15 min nach dem Aufrichten, in Verbindung mit Nackensteifigkeit, Tinnitus, Hypakusis, Fotophobie oder Nausea innerhalb von 5 Tagen nach Liquorpunktion definieren [16].

Die Inzidenz des PKS nach RA variiert in Abhängigkeit vom untersuchten Patientenkollektiv zwischen 4,6 und 11 % [5, 22]. In der Entstehung eines PKS stellt v. a. der Nadeltyp (traumatisch vs. atraumatisch) einen wesentlichen Risikofaktor dar. Die Verwendung atraumatischer Nadeln senkte die Inzidenz des PKS in verschiedenen Patientenkollektiven effektiv [5, 22]. Neben dem Nadeltyp können ein großer Nadeldurchmesser, eine horizontale Schliffausrichtung, die fehlende Mandrinreinsertion sowie anwenderassoziierte Faktoren wie mangelnde Erfahrung oder Ermüdung ebenfalls zur Entstehung eines PKS beitragen [25, 28, 29]. Zusätzlich wird eine Zunahme der Inzidenz des PKS in Abhängigkeit von Alter und Geschlecht diskutiert. Insbesondere junge Patientinnen tragen ein erhöhtes Risiko für das Auftreten eines PKS [2]. Ein niedriger Body-Mass-Index (BMI), ein chronischer Kopfschmerz oder ein bereits stattgehabter PKS in der Anamnese sind als Risikofaktoren beschrieben [3, 18]. Der PKS geht häufig mit funktionellen Einschränkungen einher und ist mit einem erhöhten Risiko für weitere neurologische Folgekomplikationen, wie z. B. dem subduralen Hämatom, assoziiert [14, 21].

Bisher gibt es keine verlässlichen Daten, die vergleichend die Inzidenz und die potenziellen Risikofaktoren für die Entstehung eines PKS bei geburtsmedizinischen und unfallchirurgischen Patient*innen untersuchen. Ziel dieser retrospektiven Datenanalyse war daher, die Inzidenz des PKS, die Therapie sowie die Auswirkungen des PKS auf die Krankenhausverweildauer zu vergleichen und gruppenspezifische Risikofaktoren für die Entstehung eines PKS in beiden Patientengruppen zu identifizieren.

## Studiendesign und Untersuchungsmethoden

Die retrospektive Datenerfassung und -analyse wurde in der Klinik für Anästhesiologie mit Schwerpunkt operative Intensivmedizin am Campus-Virchow-Klinikum der Charité – Universitätsmedizin Berlin (Ethikvotum EA 2/058/14) durchgeführt. Patientencharakteristika und Daten zum Verfahren und zum Krankheitsverlauf wurden durch Systemabfragen der Patientendatenverwaltung-Software SAP® (Fa. SAP Deutschland SE & Co. KG, Walldorf, Deutschland) und Medlinq® (Fa. Medlinq Softwaresysteme GmbH, Hamburg, Deutschland) sowie des Netzwerks für Regionalanästhesie (NRA) der Deutschen Gesellschaft für Anästhesie und Intensivmedizin (DGAI) generiert. Abfragekriterien waren die Art der RA (SPA, CSE), der Zeitpunkt der Anlage (2010–2012) und die primär behandelnde Klinik der eingeschlossenen Patient*innen (Unfallchirurgie, UCH-Gruppe; Geburtsmedizin, GEB-Gruppe). Die Datensätze wurden nach Durchsicht der Papierakten vervollständigt und anschließend pseudonymisiert ausgewertet.

Zur Beurteilung des Vorkommens eines PKS werteten 3 erfahrene Kliniker (JW, LK, CvH) Akteneinträge im postinterventionellen Verlauf nach der offiziellen Definition der International Headache Society [16] aus. Alle Patient*innen waren vor der Anlage der RA schriftlich und mündlich über das Prozedere und mögliche Komplikationen (inkl. PKS) aufgeklärt worden und regelhaft im Rahmen einer Abschlussvisite (24 h) durch den anästhesiologischen Akutschmerz- oder Kreißsaaldienst hinsichtlich eines PKS oder anderer Komplikationen nach RA untersucht worden. Patient*innen, die vor dieser Visite bereits entlassen worden waren (<1 %), galten als komplikationsfrei.

Zur statistischen Analyse wurde die Version 23.0 des SPSS-Statistik-Programms verwendet (SPSS, Inc., Fa. IBM Company, New York, USA). Die Grafiken wurden mit den Programmen Microsoft Office Excel 2013 (Fa. Microsoft Corporation, Washington, USA) und GraphPad Prism 5 (Fa. GraphPad, Kalifornien, USA) erstellt. Nach Überprüfung auf Normalverteilung mittels Kolmogorov-Smirnov-Test erfolgte eine univariate Analyse den Mann-Whitney-U-, Chi^2^- und Student’s t‑Tests mit Angabe von Median und Interquartilsabstand (IQR). Für die logistischen Regressionsanalysen wurde in beiden Gruppen in Abhängigkeit von den signifikanten Ergebnissen der univariaten Analyse für das Verfahren folgende Faktoren berücksichtigt: Alter, Köpergröße, Körpergewicht und Anlagezeitpunkt sowie zusätzlich in der geburtsmedizinischen Gruppe das regionalanästhesiologische Verfahren (CSE vs. SPA; in der unfallchirurgischen Gruppe wurden fast ausschließlich eine SPA verwendet). Dabei wurden im Gegensatz zum BMI die Köpergröße und das Körpergewicht als wichtige solitäre verfahrensrelevante Faktoren gewertet. Ein nichtsignifikanter Hosmer-Lemeshow-Test wurde für die Güte der Regressionsmodelle vorausgesetzt. Die Ergebnisse wurden als „odds ratio“ und 97,5 %-Konfidenzintervall (KI) angegeben. Als signifikant wurde eine Irrtumswahrscheinlichkeit von *p* < 0,05 für alle statistischen Tests angesehen.

## Ergebnisse

Nach Berücksichtigung der Ein- und Ausschlusskriterien konnten aus dem oben genannten Zeitraum die Daten von 341 unfallchirurgischen und 2113 geburtsmedizinischen Patient*innen ausgewertet werden.

Die Inzidenz des PKS als primärer Endpunkt betrug in der UCH-Gruppe 5,9 % (20 von 341 Patienten) und in der GEB-Gruppe 1,8 % (38 von 2113 Patientinnen).

### Unfallchirurgie

In der UCH-Gruppe wurden insgesamt 86 % American Society of Anesthesiologists (ASA) ASA-1- und ASA-2-Patienten*innen behandelt. Patient*innen, die eine PKS entwickelten gehörten zu 95% zur ASA-1- und ASA-2 Gruppe. Sowohl in der Gruppe mit als auch ohne PKS erhielten 55 bzw. 57 % der Patient*innen einen arthroskopischen Eingriff in SPA, gefolgt von Operationen am Sprunggelenk (37 %) bzw. sonstigen Eingriffen an der unteren Extremität. Als Punktionshöhe wurde in allen Gruppen der Bereich zwischen dem Lendenwirbelkörper (LWK) 3 und 4 angegeben. In allen Gruppen war eine gleichmäßige Geschlechterverteilung gegeben.

Im Vergleich der Patienten- und Verfahrenscharakteristika (Tab. 1) konnten in der univariaten Analyse signifikante Unterschiede für Alter, Gewicht und BMI zwischen den Gruppen mit und ohne PKS identifiziert werden (Tab. 1). Patient*innen mit PKS waren im Vergleich mit 38 Jahren 9 Jahre jünger als die Patient*innen ohne PKS (Tab. 1). Bei einem BMI von 23,5 kg/m^2^ und einem Köpergewicht von 70,5 kg waren sie um 1,7 kg/m^2^ im BMI bzw. 6,5 kg im Köpergewicht schlanker als Patient*innen ohne PKS (Tab. 1). Weder die Art des gewählten RA-Verfahrens, der Nadeltyp, die Nadelgröße, der Ausbildungstand (Weiterbildungsassistent/Facharzt) noch der Anlagezeitpunkt waren unterschiedlich zwischen den Gruppen (Tab. 1).

**Table 1:** 

	Unfallchirurgie (UCH)				Geburtsmedizin (GEB)			
	Total *n* = 341	PKS *n* = 20 (5,9)	Kein PKS *n* = 321	*p*-Wert	Total *n* = 2113	PKS *n* = 38 (1,8)	Kein PKS *n* = 2075	*p*-Wert
**Alter (Jahre)**	46 (34,0–62,0)	38 (26,0–45,5)	47 (34,0–62,0)	**0,011**^**c**^	32 (28,0–36,0)	31 (26,0–35,0)	31 (26,0–35,0)	0,230^c^
**Größe (cm)**	173 (165–180)	168 (161–176)	173 (165–180)	0,091^c^	163 (152–168)	163 (156–168)	163 (152–168)	0,871
**Gewicht (kg)**	76,0 (65,0–85,0)	70,5 (57,0–75,0)	77,0 (66,0–86,0)	**0,006**	78,0 (70,0–87,0)	74,5 (69,0–83,0)	78,0 (70,0–88,0)	0,156^a^
**BMI (kg/m**^**2**^**)**	25,1 (22,9–28,3)	23,5 (21,4–25,8)	25,2 (23,0–28,4)	**0,037**	26,4 (20,6–30,9)	26,5 (21,0–30,0)	26,4 (20,7–30,9)	0,725
**Verfahren**	–	–	–	0,340^b^	–	–	–	**<0,001**^**b**^
*SPA*	327 (96)	20 (100)	307 (96)		156 (7)	13 (34)	143 (7)	
*CSE*	14 (4)	0	14 (4)		1957(93)	25 (66)	1932 (93)	
**Nadelgröße (Gauge)**	27 (26–27)	27 (26–27)	27 (26–27)	0,513^a^	27 (27–27)	27 (27–27)	27 (27–27)	0,856^a^
**Nadeltyp**	*n* = 114	*n* =7	*n* = 107	0,339^b^	*n* = 1431	*n* = 29	*n* = 1402	0,812^b^
*„Pencil point“ (atraumat.)*	67 (59)	6 (86)	61 (17)		1344 (94)	27 (93)	1317 (94)	
*Facette (traumat.)*	47 (41)	1 (14)	46 (43)		87 (6)	2 (7)	85 (6)	
**Anlage**	*n* = 334	*n* = 20	*n* = 314	0,267^b^	k.A	–	–	–
*WB 1.–3.*	149 (45)	8(40)	141(45)		–	–	–	
*WB 4.–6.*	42 (12)	5 (25)	37 (12)		–	–	–	
*Facharzt*	143 (43)	7 (35)	136 (43)		–	–	–	
**Zeitpunkt**	*n* = 331	*n* = 20	*n* = 321	0,130^b^	*n* = 1606	*n* = 38	*n* = 1568	0,709
*Regel*	319 (94)	17 (85)	302 (94)		1118 (70)	28 (74)	1090 (70)	
*Dienst*	22 (6)	3 (15)	19 (6)		488 (30)	10 (26)	478 (30)	

Die Daten werden dargestellt als Median (Interquartilsabstand, IQR) oder Anzahl (Häufigkeit [%])

*PKS* postpunktioneller Kopfschmerz, *BMI* Body-Mass-Index, *KS* Kopfschmerz, *SPA* Spinalanästhesie, *CSE* kombinierte Spinalepiduralanästhesie, *WK* Wirbelkörper, *WB 1.–3*. Assistenzarzt im 1. bis 3. Weiterbildungsjahr, *Regel* Regelarbeitszeit 7–16 Uhr, *Dienst* Dienstarbeitszeit 16–7 Uhr des Folgetags, *Facette* Facettenschliff

^a^Mann Whitney U‑Test

^b^Chi^2^-Test

^c^Student’s t‑Test

Das Alter und das Köpergewicht, nicht aber die Körpergröße, konnten in der logistischen Regressionsanalyse als signifikante unabhängige potenzielle Risikofaktoren für das Auftreten eines PKS in der Unfallchirurgie identifiziert werden (Tab. 2). Hinsichtlich des Anlagezeitpunkts zeigt sich ein tendenzielles, aber nicht signifikantes, erhöhtes Risiko für einen PKS bei Anlage des Regionalverfahrens im Bereitschaftsdienst (Tab. 2).

**Table 2:** 

Risikofaktoren	OR (2,5–97,5 %-KI)	*p*-Wert
Unfallchirurgie PKS (*n* = 20)		
Alter	0,963 (0,932–0,991)	**0,015**
Köpergröße	1,000 (0,995-NA)	0,980
Köpergewicht	0,956 (0,920–0,989)	**0,014**
Anlagezeitpunkt	3,939 (0,784–15,449)	0,063
Geburtsmedizin PKS (*n* = 38)		
Alter	0,937 (0,921–1,028)	0,312
Köpergröße	1,000 (0,999–1,001)	0,788
Köpergewicht	1,001 (0,998–1,013)	0,616
Anlagezeitpunkt	1,009 (1,004-NA)	0,164
Verfahren	0,049 (0,023–0,106)	**<0,001**

Logistische Regressionsanalyse: Die Daten werden dargestellt als Mittelwert (±Standardabweichung) oder Anzahl (Häufigkeit [%]); *Fett* signifikante Ergebnisse

*PKS* postpunktioneller Kopfschmerz, *OR* Odds Ratio; *KI* Konfidenzintervall

### Geburtsmedizin

In der Geburtsmedizin wurden sowohl in der Gesamtpopulation als auch in der PKS-Gruppe bzw. der Gruppe ohne PKS zu 97 % ASA-1- und ASA-2-Patientinnen behandelt. Sowohl in der Gesamtpopulation als auch in der Gruppe ohne PKS erhielten 92 %, in der PKS-Gruppe 84 %, der Patientinnen ihr RA-Verfahren im Rahmen einer Sectio caesarea. Als Punktionshöhe wurde in allen Gruppen der Bereich zwischen dem LWK 3 und 4 angegeben.

Im Vergleich der Patienten- und Verfahrenscharakteristika in der univariaten Analyse zeigte sich, bei insgesamt homogenerer Altersverteilung im Vergleich zum unfallchirurgischen Kollektiv, kein signifikanter Zusammenhang von Alter, BMI und der Inzidenz des PKS (Tab. 1). Hinsichtlich der Art des RA-Verfahrens wurde zwischen den Gruppen mit und ohne PKS eine signifikant niedrigere Inzidenz des PKS bei Patientinnen mit einer CSE (1,2 %) im Vergleich zur SPA (8,3 %) gefunden (Tab. 1).

Weder der Nadeltyp, die Nadelgröße, noch der Anlagezeitpunkt waren unterschiedlich zwischen den Gruppen (Tab. 1).

Die Art des RA-Verfahrens (SPA vs. CSE) wurde in der logistischen Regression als möglicher unabhängiger Risikofaktor für einen PKS bestätigt (Tab. 2). Das Alter, die Köpergröße, das Köpergewicht und der Anlagezeitpunkt stellten keine Risikofaktoren in der Entstehung eines PKS dar (Tab. 2).

### Therapie

In der Therapie des PKS unfallchirurgischer Patienten wurde in den meisten Fällen eine konservative Therapie (Lagerungsmaßnahmen, Flüssigkeitsbilanzierung, symptomatische Therapie mit Analgetika und Antiemetika) in Verbindung mit Koffein verabreicht (Tab. 3). Nur 2 Patient*innen erhielten einen epiduralen Blut-Patch nach mindestens 24-stündiger konservativer Therapie (Tab. 3). Die mediane, stationäre Therapiedauer betrug 3 Tage (Tab. 3).

**Table 3:** 

	GEB-Gruppe (*n* = 38)	UCH-Gruppe (*n* = 20)	*p*-Wert
*Therapie PKS*	*–*	*–*	*0.007* ^a^
*Konservativ*	11 (29)	5 (25)	**–**
+Koffein	10 (26)	13 (65)	
*Invasiv/EBP <24* *h*	0 (0)	0 (0)	
*Invasiv/EBP >24* *h*	17 (45)	2 (10)	
*Therapiedauer (Tage)*	2 (2–4)	3 (2,75–4,25)	0.127 ^a^

Die Daten werden dargestellt als Median (Interquartilsabstand, IQR) oder Anzahl (Häufigkeit [%])

*PKS* postpunktioneller Kopfschmerz, *EBP* epiduraler Blut-Patch, konservativ (Lagerungsmaßnahmen, Flüssigkeitsbilanzierung, symptomatische Therapie mit Analgetika und Antiemetika)

^a^Chi^2^-Test

Im Vergleich zur UCH-Gruppe wurden Patientinnen mit PKS der GEB-Gruppe signifikant häufiger mit einem epiduralen Blut-Patch nach 24 h (Tab. 3), begleitet von einer konservativen Therapie (Lagerungsmaßnahmen, Flüssigkeitsbilanzierung, symptomatische Therapie mit Analgetika und Antiemetika) und Koffein, behandelt (Tab. 3). Die Therapiedauer war im Median mit 2 Tagen nicht signifikant kürzer als in der UCH-Gruppe (Tab. 3).

### Krankenhausverweildauer

**Figure 1:**
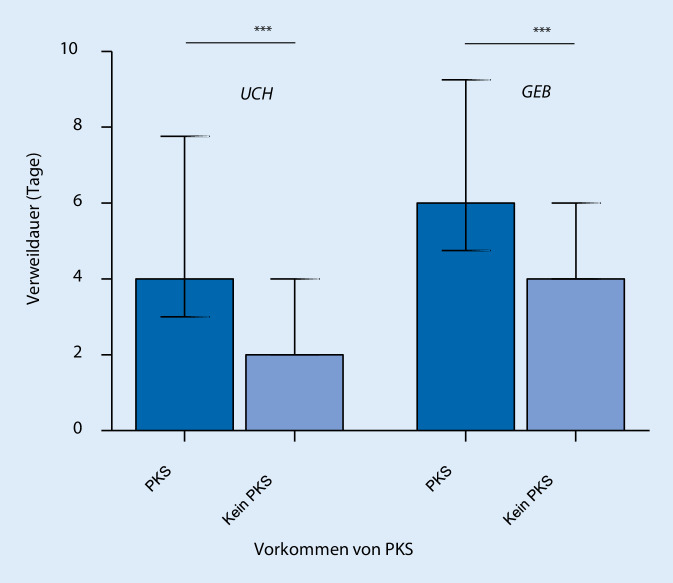

In der UCH-Gruppe war die mediane Krankenhausverweildauer der Patient*innen mit PKS mit 4 Tagen (IQR 3 bis 7,8 Tage) um 2 Tage signifikant länger als bei Patient*innen ohne PKS (IQR 2 bis 4 Tage) (Abb. 1). Auch in der GEB-Gruppe war die mediane Verweildauer für Patientinnen mit PKS mit 6 Tagen (IQR 4,8 bis 9,3 Tage) um 2 Tage signifikant länger als bei Patientinnen ohne PKS (IQR 4 bis 6 Tage) (Abb. 1).

## Diskussion

Zu Inzidenz, Risikofaktoren und Outcome des PKS bei unfallchirurgischen und geburtsmedizinischen Patienten liegen nur wenige vergleichende Daten vor. In unserer Untersuchung betrug die Inzidenz des PKS 5,9 % in der UCH-Gruppe sowie 1,8 % in der GEB-Gruppe. Signifikante Risikofaktoren waren in der UCH-Gruppe ein jüngeres Patientenalter und ein geringeres Körpergewicht. In der GEB-Gruppe war hingegen die Art des RA-Verfahrens (SPA vs. CSE) signifikant mit dem Auftreten eines PKS assoziiert. In beiden Gruppen führte der PKS zu einer signifikant verlängerten Krankenhausverweildauer um 2 Tage.

Die von uns beobachtete höhere Inzidenz des PKS in beiden Patientengruppen ist mit den Angaben aus der Literatur vergleichbar, in der für die UCH-Gruppe eine Häufigkeit zwischen 0 und 17,6 % [15] und für die GEB-Gruppe von 0 bis 6,5 % [5] angegeben werden.

Den größten Einfluss auf die Entstehung eines PKS scheinen die Nadeleigenschaften zu haben [5, 15]. In einem aktuellen systematischen Review mit Einschluss von über 30.000 Patient*innen wird gezeigt, dass die Verwendung atraumatischer Spinalnadeln bei gleicher Effektivität zu einem signifikant geringeren Auftreten an PKS führt [22]. Auch wenn in unserer Untersuchung ein signifikanter Zusammenhang zwischen PKS und Nadeltyp in beiden Gruppen nicht gezeigt werden konnte, könnte die häufigere Verwendung traumatischer Nadeln in der UCH-Gruppe (41%) im Vergleich zur GEB-Gruppe (6 %) die höhere Inzidenz des PKS in der UCH-Gruppe erklären. Unsere Daten stützen somit die Daten aus der Literatur, die mit Verwendung atraumatischer Nadeln eine Senkung der PKS-Inzidenz zeigen [5, 29, 30].

Obwohl bei geburtsmedizinischen Patientinnen 2 bedeutende Risikofaktoren (junges Alter und weibliches Geschlecht) häufiger vertreten waren [24], wiesen Patientinnen in der GEB-Gruppe eine niedrigere Inzidenz des PKS als die unfallchirurgischen Patienten auf. Betrachtet man jedoch nur die Gruppe der Spinalanästhesien in der GEB-Gruppe, so war die Inzidenz des PKS vergleichbar der UCH-Gruppe. Geburtsmedizinisch-anästhesiologische Studien untersuchten bislang hauptsächlich die akzidentelle Duraperforation bei Periduralanästhesien (ADP) zur Schmerztherapie bei der vaginalen Geburt [18]. Nach ADP ist die Inzidenz eines PKS, wahrscheinlich aufgrund des vermehrten Liquorverlusts durch Pressen während der Geburt und des größeren Traumas durch die Tuohy-Nadel, mit 50–88 % wesentlich höher [8]. Die Vergleichbarkeit der Daten mit unserer Analyse ist nur bedingt möglich, da unsere Untersuchung hauptsächlich SPA zur Sectio caesarea einschloss, die primär im Rahmen der kombinierten Spinalepiduralanästhesie oder als alleinige SPA zum Einsatz kamen. Dabei spricht in unseren Daten die niedrigere Inzidenz des PKS nach CSE im Vergleich zur SPA für die Sicherheit der CSE trotz des Risikos einer akzidentellen Duraperforation. Jedoch muss dabei in Betracht gezogen werden, dass die geburtsmedizinischen Patientinnen vor Entfernung des Periduralkatheters (2 h postoperativ vor Entlassung der Patientinnen aus dem Aufwachraum) 3 mg Morphin epidural (mit 0,9 %iger Natriumchloridlösung auf 10 ml verdünnt) zur postoperativen Analgesie erhielten. Die prophylaktische, epidurale Morphingabe reduzierte in der prospektiven, randomisierten und doppelt verblindeten Studie von Al-Metwalli 2008 [1] die Häufigkeit des PKS und könnte somit eine Erklärung für das geringere Auftreten des PKS nach CSE erklären. 

Verschiedene Risikofaktoren, die das Auftreten eines PKS begünstigen, sind in der Literatur beschrieben. Hierzu zählen z. B. das Alter, Geschlecht, BMI bzw. das Köpergewicht, Eigenanamnese, Nadeltyp, Nadelgröße, Schliffausrichtung, Mandrinreinsertion, Erfahrungsstand und die Ermüdung des Anästhesisten [6, 7]. Dabei wurde ähnlich der UCH-Gruppe unserer Untersuchung ein höheres Risiko für das Auftreten eines PKS bei jungen und schlanken Frauen [12] sowie bei hoher Arbeitsbelastung oder Ermüdung des Arztes während des Bereitschaftsdienstes [29] beschrieben. Die Tatsache, dass bei geburtsmedizinischen Patientinnen, bei denen die Anlage der RA zur Sectio caesarea regelhaft häufiger außerhalb der Regelarbeitszeit erfolgt, die Anlage im Bereitschaftsdienst jedoch nicht als Risikofaktor für die Entstehung eines PKS identifiziert werden konnte, ist möglicherweise dem höheren Ausbildungsstand der Anästhesist*innen (>3. Weiterbildungsjahr) zu schulden, die regelhaft im Kreißsaal tätig sind.

Weitere in der Literatur beschriebene Risikofaktoren, wie beispielsweise die Nadelgröße [10, 11], wurden in unserer Untersuchung nicht bestätigt. Hinsichtlich der Nadelgröße könnte dies an dem Umstand liegen, dass in beiden Gruppen die überwiegend verwendete Nadelgröße einen Durchmesser von 27 G aufwies. Dies könnte bestätigen, dass zur Prävention des PKS mittlerweile andere Faktoren in den Vordergrund rücken und die Nadelgröße, sofern kleine Nadelgrößen von 25–27 G, wie in der Literatur empfohlen, benutzt werden [6], einen zu vernachlässigenden Risikofaktor darstellt. Auch konnten aufgrund der Homogenität der Patientencharakteristika in der geburtsmedizinischen Gruppe z. B. Alter, Köpergewicht und Geschlecht in dieser Gruppe nicht als Risikofaktoren identifiziert werden.

Die Therapie des PKS unterschied sich in den untersuchten Patientengruppen deutlich. In der UCH-Gruppe stand die erweiterte medikamentöse Therapie (konservative Therapie, inkl. Analgetika und Koffein) im Vordergrund. In der GEB-Gruppe hingegen wurde der epidurale Blut-Patch fast ebenso häufig durchgeführt wie die erweiterte medikamentöse Therapie. Dies mag dem Umstand geschuldet sein, dass die RA im Vergleich zur Allgemeinanästhesie das führende anästhesiologische Verfahren ist und somit das pflegerische und ärztliche Personal auf den Wochenbettstationen im Umgang mit PKS geschult und der Zusammenhang von rückenmarknahen Punktionen und PKS besser bekannt ist. Vor allem aber sorgt vermutlich ein höherer Leidensdruck der Patientinnen, bedingt durch die psychische Belastung (Einschränkung in der Versorgung des Neugeborenen) und ein mögliches Stilldefizit, für eine offensivere Anwendung des epiduralen Blut-Patch zur Therapie des PKS in der Geburtsmedizin.

Es ist nicht auszuschließen, dass es aufgrund des insgesamt selteneren Einsatzes der RA in der Unfallchirurgie zu einer Fehleinschätzung des PKS kommen kann, insbesondere dann, wenn diese erst am zweiten oder dritten Tag nach Anlage der SPA auftreten. Gegebenenfalls können zukünftig nach sorgfältiger Abwägung der Risiken auch neuere Therapieverfahren wie z. B. die Blockade des Ganglion sphenopalatinum oder zervikale Nervenblockaden eine zunehmende Rolle spielen [9, 17], obwohl diese nicht die pathophysiologische Ursache des PKS behandeln, sondern eine symptomorientierte Schmerztherapie darstellen.

In beiden untersuchten Patientengruppen zeigte sich bei Auftreten eines PKS eine um 2 Tage verlängerte Krankenhausverweildauer. Dieser Zusammenhang wurde bereits in vorhergehenden Untersuchungen gezeigt [4, 23]. Inwieweit dieser abhängig vom Schmerzverlauf des PKS und von möglichen begleitenden PKS-Symptomen ist, bleibt ungeklärt, wobei v. a. in der Geburtsmedizin mögliche PKS-bedingte funktionelle Einschränkungen der Mutter die Versorgung ihres Neugeborenen einschränken können [19]. Neben der Belastung der Familie durch eine verlängerte Liegedauer und die damit verzögerte Entlassung in die häusliche Umgebung fallen durch die längere Liegezeit nichtunerhebliche Mehrkosten für den Krankenhausträger an, welche durch eine frühzeitige Diagnostik und Therapie des PKS evtl. vermeidbar wären.

## Limitationen

Die vorliegende Untersuchung wird durch ihren retrospektiven Charakter und die damit möglichen Einschränkungen der Datenqualität limitiert. Zudem erlauben retrospektive Untersuchungen keine kausalen Erklärungen zwischen Intervention und Komplikation, sondern zeigen lediglich statistische Zusammenhänge auf. Diese können Hypothesen als Grundlage prospektiver Untersuchungen generieren. Ferner sind bei der vergleichenden Interpretation der Ergebnisse der beiden Patientengruppen Unterschiede in Gruppengröße, Alter und Geschlecht und Art der Operation zu beachten. Daten zur Kopfschmerzanamnese bzw. zur Migräneanamnese oder zu einem PKS bzw. Blut-Patch im Rahmen eines früheren RA-Verfahrens waren retrospektiv nur unvollständig zu erheben. Der PKS als Folge einer akzidentellen Duraperforation im Rahmen einer Periduralanästhesie war aufgrund fehlender Daten nicht Gegenstand dieser Untersuchung.

## Schlussfolgerung

In der vorliegenden Untersuchung wurden die Inzidenz, potenzielle Risikofaktoren, Therapie sowie der Einfluss des PKS auf die Krankenhausverweildauer an 2 großen Patientenkollektiven der Charité – Universitätsmedizin Berlin analysiert. Im Vergleich zur Literatur war die Inzidenz des PKS bei unfallchirurgischen und geburtsmedizinischen Patient*innen gering. Die überwiegende Verwendung atraumatischer Nadeln mit kleinen Nadelgrößen (≤25 G) in beiden Gruppen könnte ursächlich für die insgesamt niedrige Inzidenz des PKS sein. Je nach Kollektiv konnten unterschiedliche Risikofaktoren, wie z. B. ein junges Alter, eine schlanke Konstitution sowie die Wahl des Verfahrens identifiziert werden und Daten der Literatur zur Risikofaktorenanalyse des PKS bestätigen.

Die schwere Beeinträchtigung des Allgemeinbefindens der Patient*innen mit PKS und dessen Therapie verlängerte die durchschnittliche Krankenhausverweildauer in beiden Gruppen. Dies richtet den Fokus darauf, dass zukünftige Studien eine schnelle Diagnostik und ursachengerechte Behandlung des PKS untersuchen sollten. Schulungen in Bereichen mit einem geringen Anteil an rückenmarknahen Verfahren können zum frühzeitigen Erkennen eines PKS und dem zeitnahen Beginn einer konservativ-medikamentösen oder epiduralen Blut-Patch-Behandlung führen. Entsprechend unseren Daten könnten die Krankenhausverweildauer gesenkt, weitere Komplikationen verhindert [14] bzw. die Patientenzufriedenheit im Wochenbett verbessert werden.

## Fazit für die Praxis

- Die Inzidenz der Komplikation postpunktioneller Kopfschmerz (PKS) ist in der untersuchten unfallchirurgischen und geburtsmedizinischen Klientel niedrig.
- Jüngere und schlanke Patienten mit vermeintlich einfacheren Punktionsbedingungen tragen möglicherweise ein erhöhtes Risiko für einen PKS.
- Kosten für das Gesundheitssystem könnten wahrscheinlich durch eine Verkürzung der Krankenhausverweildauer gesenkt werden.
